# Genetic risk factors of ME/CFS: a critical review

**DOI:** 10.1093/hmg/ddaa169

**Published:** 2020-08-03

**Authors:** Joshua J Dibble, Simon J McGrath, Chris P Ponting

**Affiliations:** 1 MRC Human Genetics Unit, Institute of Genetics and Molecular Medicine, University of Edinburgh, Crewe Road South, Edinburgh EH4 2XU, UK; 2 Wrekin, Prospect Road, Monmouth NP25 3SZ, UK

## Abstract

Myalgic encephalomyelitis/chronic fatigue syndrome (ME/CFS) is a complex multisystem illness that lacks effective therapy and a biomedical understanding of its causes. Despite a prevalence of ∼0.2–0.4% and its high public health burden, and evidence that it has a heritable component, ME/CFS has not yet benefited from the advances in technology and analytical tools that have improved our understanding of many other complex diseases. Here we critically review existing evidence that genetic factors alter ME/CFS risk before concluding that most ME/CFS candidate gene associations are not replicated by the larger CFS cohort within the UK Biobank. Multiple genome-wide association studies of this cohort also have not yielded consistently significant associations. Ahead of upcoming larger genome-wide association studies, we discuss how these could generate new lines of enquiry into the DNA variants, genes and cell types that are causally involved in ME/CFS disease.

## Introduction

Myalgic encephalomyelitis/chronic fatigue syndrome (ME/CFS) is a long-term, multisystem illness of unknown aetiology whose symptoms are highly debilitating (1). Large numbers of individuals are affected, estimated at 0.2% of the UK population (2), with the ratio of women to men diagnosed as high as 4:1 (2,3). This results in a substantial annual economic burden assessed at ∼£3.3 billion annually in the UK (https://meassociation.org.uk/wp-content/uploads/2020Health-Counting-the-Cost-Sept-2017.pdf). Those diagnosed with ME/CFS experience diverse physical and cognitive symptoms but are most distinguished on the basis of post-exertional malaise, defined as a substantial worsening of symptoms following mental or physical activity that to healthy individuals would be minor, and which are not alleviated by resting (1,4). The quality of life of people with ME/CFS is worse than for many other illnesses, including some cancers (5).

Individuals with ME/CFS can be diagnosed after failing to recover fully following acute infection from viral and non-viral pathogens (6). In such cases it is assumed that infection initiates ME/CFS and thus contributes to its aetiology. Overall, however, the biological basis to ME/CFS is poorly understood, and currently there is little consensus among investigators on the molecular, cellular and genetic factors that alter risk of ME/CFS (7). Many biomolecular studies have sought biomarkers (factors such as RNAs, microRNAs, metabolites or proteins) whose abundance in blood, for example, accurately and reproducibly distinguishes ME/CFS cases from controls (8–14). Unfortunately, such a factor has yet to be identified in multiple independent studies. If it were to be found then it would also need to accurately separate ME/CFS cases from people with unrelated diseases.

Multiple members of the same family can be diagnosed with ME/CFS (15,16), which implies that inherited (i.e. genetic) factors might contribute to ME/CFS risk. However, ME/CFS diagnoses do not follow a predictable Mendelian pattern, which reveals that there is not one genetic variant that increases ME/CFS risk. Instead, ME/CFS is likely to be a complex multifactorial disorder whose genetic contributions are many and varied, as is known to be the case for example for many autoimmune diseases (17). These varied contributions will likely be of small individual effect, but together disrupt many genes and cellular or physiological processes. Each such variant is expected to contribute little to altered risk, implying that even in aggregate they cannot provide a reliable diagnosis. Genetics can objectively identify genes, molecules and cellular pathways that contribute to ME/CFS genetic risk, and these can then be targeted therapeutically. By contrast, molecular differences observed between patients and controls in other biomolecular studies could reflect the many secondary consequences of disease (e.g. a more sedentary lifestyle) rather than the disease’s primary cause.

Here we first summarize ME/CFS diagnosis criteria before detailing the evidence for ME/CFS heritability and genetic risk factors. We then review the methodology, limitations and advantages of genome-wide association studies as applied to ME/CFS. This review is timely because it precedes the launch of DecodeME (www.decodeme.org.uk) and a similar study from the University of Oslo (www.ukbiobank.ac.uk/tag/cfs-me/).

## ME/CFS Case Definitions

Population genetics studies of disease rely on accurate case definition. For ME/CFS, however, no laboratory diagnostic test is currently approved. Rather, clinical diagnosis is made on the basis of physical examination, case history and exclusion of other disorders. For research studies, investigators do not currently apply a single set of diagnostic criteria for ME/CFS or CFS cases. Of the 20 existing sets of diagnostic criteria or case definitions (1), three are commonly applied in research and the use of a fourth (US Institute of Medicine, now called the National Academy of Medicine [IOM/NAM]) is expected in the future (Box 1). These different diagnostic criteria will select distinct, albeit overlapping, case cohorts and thus may not yield equivalent biomolecular findings.

Box 1 ME/CFS Case Inclusion/Exclusion Criteria

1) The Fukuda Criteria developed in 1994 by the International Chronic Fatigue Syndrome Study Group (18). To be diagnosed with CFS, a person must display unexplained, persistent or relapsing chronic fatigue, as well at least four of eight additional symptoms.
2) The Canadian Consensus Criteria (19). To be diagnosed, people with ME/CFS must exhibit a broad array of symptoms in specific combinations, namely:
  - cardinal symptoms of fatigue, post-exertional malaise or post-exertional fatigue, sleep dysfunction and pain (myalgia, often including headaches);
  - two or more neurological and/or cognitive symptoms from a given list;
  - at least one symptom from two of the following categories: autonomic, neuroendocrine and immune manifestations (with a list of relevant symptoms provided for each) and
  - symptoms must have persisted for at least 6 months.
  The authors also provided a list of co-morbidities and another of illnesses that exclude the diagnosis of ME/CFS. These criteria seek to define patients with diverse symptoms and have been proposed to preferentially select individuals with more severe symptoms (20).
3) International Consensus Criteria (21): This modifies the Canadian Consensus Criteria by revising criteria, adding others and removing both chronic fatigue as a criterion and the requirement of a 6-month period prior to diagnosis. For diagnosis, a patient must display:
  - post-exertional neuroimmune exhaustion (broadly equivalent to post-exertional malaise);
  - three neurological impairment symptoms—at least one each from three of four categories;
  - three immune, gastrointestinal or genitourinary symptoms—at least one from three of five categories and
  - atleast one energy production/transportation symptom.
4) IOM/NAM Criteria developed for clinical not research purposes (1). Criteria were streamlined and focussed on core symptoms:
  - substantial reduction from pre-illness activity levels, evidence of post-exertional malaise and unrefreshing sleep and
  - cognitive impairment and/or orthostatic intolerance.

## Evidence That CFS Risk Is Inherited

Various observations are consistent with genetic factors contributing to CFS risk for some individuals. Individuals with a CFS diagnosis (Fukuda or ICD-9 code 780.71 criteria) have a significant excess relatedness over the wider population for both close (first- or second-degree) and distant (third-degree) relatives (16,22). Of three studies that have estimated narrow-sense heritability (*h^2^*) using large cohorts, two reported non-zero *h^2^*-values that provide evidence for heritability of risk for CFS and, presumably, ME/CFS. An analysis of US health insurance claimed a high narrow-sense heritability (${h}^2=0.48$) of CFS (23), whereas an analysis of the UK Biobank individuals self-reporting a CFS diagnosis reported a less striking heritability (single nucleotide polymorphism- [SNP-] based approximate *h^2^* = 0.08 with low confidence) (24) (http://www.nealelab.is/uk-biobank). The third, a large twin-based study of CFS-like cases, produced an inconclusive result, with the 95% confidence interval of *h^2^* including zero [0.03 (0.00–0.65)] (25).

## Mitochondrial and Human Leukocyte Antigen Genetics

Independent studies confirm that clinically proven mitochondrial DNA (mtDNA) variants do not commonly explain ME/CFS (26–28). People with ME/CFS, however, appear more likely to carry mtDNA that lacks even mildly deleterious variants (28), a finding whose implications require further investigation.

Human leukocyte antigen (HLA) proteins enable the immune system to differentiate self- from non-self-cells such as foreign pathogens. Their genes exhibit extreme population polymorphism and certain HLA types are genetically predisposed to particular autoimmune diseases (29). Two independent HLA types tagged by HLA-C*07:04 or HLA-DQB1*03:03 were recently shown to be significantly associated with ME/CFS status (Canadian consensus criteria) (30). These alleles are each carried by ~ 10% of ME/CFS individuals and alter risk by ∼1.5–2.0-fold. If these results are independently replicated then they indicate that genetic differences in the human immune system alter risk for ME/CFS.

## Genome-Wide Association Study

A genome-wide association study (GWAS) is ideal for discovering genetic causes of disease and new biology particularly when disease aetiology is unknown, as is the case for ME/CFS. This is not just because it is comprehensive but because its results are not influenced by pre-existing biological assumptions or hypotheses. GWAS was central to unexpected findings, for example of the role of glia, rather than neurons, in Alzheimer’s disease pathogenesis (31) and of components of the interleukin-23 pathway in ankylosing spondylitis, psoriasis and psoriatic arthritis (32). Despite GWAS being expensive, sales of medications that have benefitted from this method already exceed its costs (32) which anyway are declining rapidly.

In a GWAS ∼.5–2.5 million SNPs are genotyped and a stringent threshold of the test statistic (*P*-value) applied: < 5 × 10^−8^ for SNPs with minor allele frequency (MAF) > 5%, or < 1×10^−8^ for MAF ≥ 0.1% (33,34). For copy number variants (CNVs) the *P*-value threshold is less stringent because they are fewer in number, although no broad consensus on its value has yet been reached. To be adequately powered to find DNA variants of small effect, GWAS requires SNP genotype data from large numbers of cases and controls (preferably from many more controls than cases). Case sample sizes of ∼10^2^ are well powered only to identify common (MAF ~ 10%) variants with very strong effects (effect size *β* ~ 1), whereas sample sizes of 10^4^ are required to identify MAF ~ 10% variants with weaker effects (*β* ~ 0.1) (32). This explains why GWAS often employ cohort sizes of ~10^4^–10^6^, and why when studies increase their cohort sizes they tend to discover larger numbers of lower-effect loci (35).

## ME/CFS Genetics and the UK Biobank

The UK Biobank is a 500 000-strong cohort that has been genotyped and exceptionally well phenotyped and hence is well suited to GWAS (36). One of the over 750 anthropometric and disease-related traits captured by the UK Biobank is self-reported clinical diagnosis of CFS. This generated the largest cohort of people collected to date who have self-reported a clinical diagnosis (1829 people). This number was ∼0.45% of all the UK Biobank participants and showed a marked female bias (0.61% of females and 0.26% of males), as expected (37). As there is a likely ‘healthy volunteer’ selection bias (38) in the UK Biobank, these prevalence estimates are lower-bound values. It is unknown how many of these 1829 participants meet the ME/CFS criteria above; hence, all results discussed below are preliminary and, as ever, require statistically robust replication.

**Table 1:** Summary of SNPs identified as significant in the UK Biobank CFS Cohort

DNA variant chromosome nearby gene			Minor allele freq (gnomAD)	*P* (Neale) female	*P* (Neale) male	*P* (Neale) both	*P* (SAIGE) both	*P* (GeneAtlas) both	*P* (Global Biobank Engine)	*P* (Pan-UKBB)
rs7337312	13	*SLC25A15*	0.54	**2.6 × 10** ^**−8**^	0.74	4.0 × 10^−6^	0.29	0.00017	9.3 × 10^−6^	2.1 × 10^−4^
rs150954845	10	*P4HA1*	0.00029	Not reported	Not reported	Not reported	0.37	**2.6 × 10** ^**−12**^	Not reported	Not reported
rs148723539	10	*EBF3*	0.0093	9.7 × 10^−7^	6.1 × 10^−4^	**2.3 × 10** ^**−9**^	0.29	Not reported	Not reported	3.7 × 10^−6^
rs564809936	4	*COX7B2*	0.00016	Not reported	Not reported	Not reported	**3.8 × 10** ^**−8**^	Not reported	Not reported	4.8 × 10^−3^

Bold font indicates nominally significant *P*-values (<5 × 10^−8^). These five studies are cited in the main text.

Five groups have performed a case–control GWAS on CFS cases in the UK Biobank. Unfortunately, they reach no consensus and their results are far from being definitive. One study found no DNA variant to pass the *P* < 5 × 10^−8^ threshold (39). Another study reported results both partitioned and unpartitioned by sex (http://www.nealelab.is/uk-biobank) finding one variant associated with female CFS status and another with male or female CFS status (Table 1). Two other studies (24,40) highlighted a single variant each (Table 1). The final study (https://pan.ukbb.broadinstitute.org/) found no variants associated with either the Biobank CFS phenotype (Table 1), or the ‘phecode’ definition of CFS (41). Consequently, despite these five studies analyzing the same data set, not a single associated DNA variant was replicated by multiple analyses. Two explanations of this lack of replication are likely. First, as alleles become rarer the likelihood increases that all people with the minor allele are placed among the cases just by chance. Two of the highlighted SNPs that are very rare in the population (MAF < 0.5%) are possible examples of this. Second, DNA sites that appear to have three or more alleles are usually excluded from analyses because they likely reflect technical genotyping artefacts (http://www.ukbiobank.ac.uk/wp-content/uploads/2014/04/UK-Biobank-Axiom-Array-Content-Summary-2014-1). One of the highlighted variants (rs148723539) is one such multi-allelic site (Table 1).

**Figure 1:**
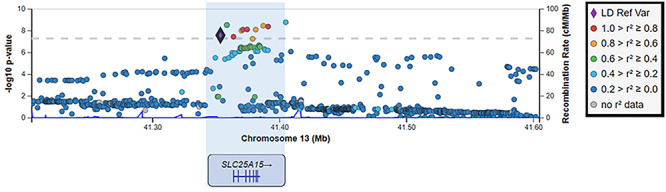
A ME/CFS-associated GWAS locus on chromosome 13 (X-axis). Genome-wide significant SNPs from Neale *et al*. http://www.nealelab.is/uk-biobank are those lying above the horizontal dashed line. The location of the *SLC25A15* gene is indicated below (exons are shown as vertical bars). The left-hand Y-axis reflects the statistical confidence (−log10 *P*-value) of the association between DNA variant and ME/CFS case status. The right-hand Y-axis (blue data curves) indicates the low extent of recombination within this locus. rs7337312 is highlighted as the reference variant (purple diamond). The degree of linkage disequilibrium of rs7337312 with neighbouring SNPs is indicated as *r*^2^ (right-hand legend).

The single remaining variant (rs7337312) is neither rare (MAF ≈ 0.5) nor multi-allelic and was identified in the female-only CFS GWAS by Neale *et al.* (http://www.nealelab.is/uk-biobank). This variant, together with adjacent significant variants that are in linkage disequilibrium, occur within a 51 kb region containing the *SLC25A15* gene (Fig. 1). In most cases, the gene affected by DNA variation is not the nearest (42) because of long-range genetic regulation (43). Nonetheless, because the rs7337312 variant predicts the amount of *SLC25A15* mRNA transcribed from this gene in some tissue samples, *SLC25A15* could be a causal gene of altered CFS risk. *SLC25A15* encodes the Ornithine Transporter type 1 protein that transports ornithine (as well as lysine and arginine) across the inner membrane of mitochondria to the mitochondrial matrix. Ornithine is an amino acid that plays a role in the urea cycle. A person with the rs7337312 CFS risk allele is expected to produce lower amounts of *SLC25A15* mRNA resulting in reduced transport of ornithine into the mitochondrion and higher amounts of ornithine in blood. Yamano *et al.* (44) and Naviaux *et al.* (14), but not Armstrong *et al.* (45), report some evidence in support of this prediction.

Using the same UK Biobank data, Aguirre *et al.* (46) tested for association with CNVs, finding two genes (*TCOF1* and *THUMPD2*) to have a greater number of CNVs (either gains or losses) in the UK Biobank CFS cases than in controls after applying a multiple-testing significance threshold of *P* < 3.1 × 10^−6^. Nevertheless, these results need to be treated with caution owing to CNV calling artefacts being prevalent at the low allele frequencies (<0.1%) used in the study.

## Studies Not Using the UK Biobank Data

Smith *et al*. (47) undertook a GWAS on CFS (defined using Fukuda criteria) for very low numbers (40 cases and 40 non-fatigued control subjects) and reported 65 DNA variants as being associated at a non-standard highly permissive threshold of *P* < 10^−3^. When the well-established threshold of *P* < 5 × 10^−8^ is applied, however, none remain significant. This is expected because, as discussed above, GWAS using this number of cases are only well powered to identify population-frequent alleles with strong effects.

**Figure 2:**
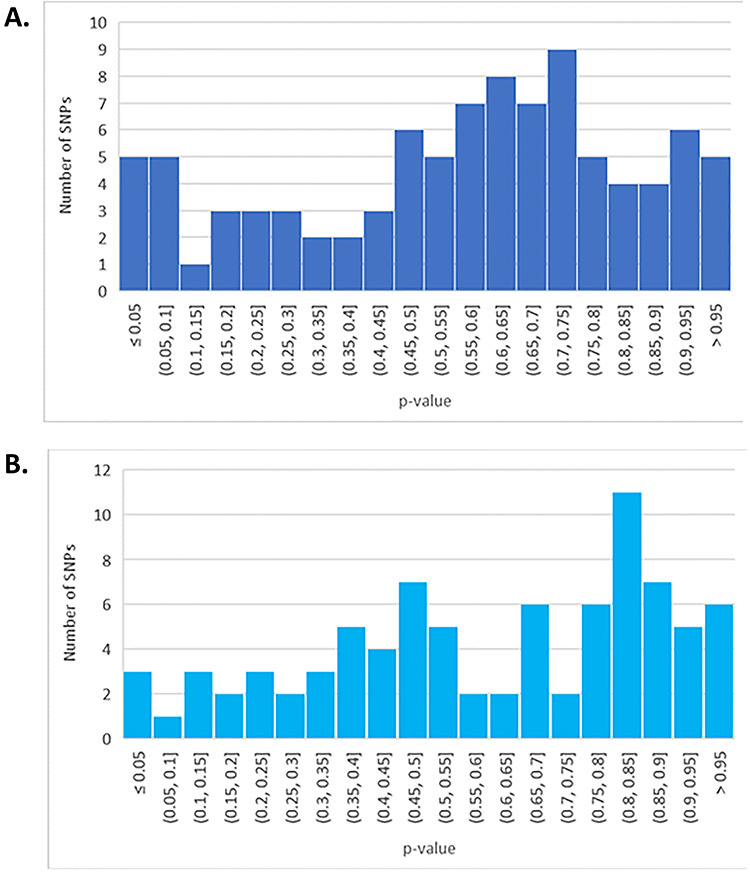
Histograms of replication *P*-values for association of SNPs for CFS risk. These *P*-values were obtained from the UK Biobank CFS GWAS (24). Only variants tested in the UK Biobank were considered. (**A**) Association *P*-values for SNPs identified in reference (59). (**B**) Association *P*-values for SNPs identified in references (60) and (52).

A second GWAS also used similarly small cohort sizes (42 cases, defined using both Canadian and Fukuda criteria, and 38 controls) and reported 299 DNA variants as associated with ME/CFS status at *P* < 1 × 10^−5^ (48). The authors justified this permissive threshold as being an inclusion criterion of the GWAS Catalogue (49). Nevertheless, *P* < 1 × 10^−5^ is used by the GWAS Catalogue for reporting purposes only, and only for results from the overall (initial GWAS plus replication) population, when this study lacked a replication cohort. Fifteen variants were reported as genome-wide significant at *P* < 5 × 10^−8^, but these associations are not replicated in the UK Biobank cohort (Fig. 2).

Perez *et al*. (50) conducted a genetics study using 383 people who self-reported a clinical diagnosis of ME/CFS (Fukuda criteria). The study only considered DNA variants that disrupted genes despite >90% of variants associated with diseases or traits lying elsewhere in the genome (51). Using non-standard thresholds on variant frequency, the authors reported 5693 DNA variants that are 2-fold more (or less) frequent in cases versus controls. However, such case–control allele frequency inequalities could have two technical, rather than biological, explanations. Firstly, errors stemming from poor DNA sample quality, incomplete DNA hybridization to the genotyping array or poorly performing array probes. Controlling for these errors imperfectly, or inconsistently between case and control genotypes, has led to retraction of publications reporting genetic associations (for example, (52)). Secondly, errors arising because confounding effects from the controls such as differences in genetic ancestry were not accounted for.

## Candidate Gene Studies

A recent review by leading human geneticists stated that: ‘initial efforts targeting variants within “candidate” genes were plagued by inadequate power, unduly liberal thresholds for declaring significance and scant attention to sources of bias and confounding, resulting in overblown claims and failed replication’ (53). Their confidence stems from knowledge that tens of thousands of associations have been identified by GWAS that subsequently are often replicated independently (49). An example of a candidate gene approach in ME/CFS research is a study of nine DNA variants in the gene *NR3C1* for 40 people with CFS and 42 controls (54). Four of these variants (rs1866388, rs2918419, rs860458 and rs6188) passed their statistical threshold for significance (*P* < 0.05). Nevertheless, none of these variants survive a Bonferroni multiple testing correction (*P* < 0.05/9 or <0.006) and none are replicated with the UK Biobank CFS cohort (24) (*P* = 0.96, 0.71, 0.71 and 0.97, respectively).

To generalize this point, we again exploited the large cohort of self-reported CFS participants of the UK Biobank. We tested for replication a large set of genetic findings reported in 16 CFS studies published between 2003 and 2015 that met the systematic review criteria of Wang *et al.* (55). Looking up associations between DNA variants (Table 1 of reference (55)) and CFS status in the UK Biobank (24) for the 11 studies that had readily available SNP references yielded a *P*-value distribution between 0 and 1 (Fig. 2A). Replication would require these *P*-values to be skewed towards small values. No such skew is observed (Fig. 2A), consistent with random samples; hence, these initial findings show no evidence of being replicated.

This conclusion is further substantiated from plotting the replication *P*-values for a further set of 77 DNA variants from Marshall-Gradisnik *et al.* (56), and 23 from Schlauch *et al.* (48): these *P*-values also show a lack of skew towards small values (Fig. 2B).

## Expected Outcomes of a ME/CFS GWAS

GWAS are proposed to have ‘substantially improved our understanding of the mechanisms responsible for many rare and common diseases and driven development of novel preventative and therapeutic strategies’ (53). This suggests that large GWAS on ME/CFS are overdue. Replicated results from such studies would have four important implications.

Firstly, it would catalyze the gain of much-needed insight into genes, cellular processes and tissues or cell types that causally alter risk for ME/CFS. When combined with functional genomics and other technologies (53), a well-designed GWAS can pinpoint multiple chromosomal locations containing DNA variants that change the activity of genes—in specific cells or tissues—which thereby alter a person’s risk of ME/CFS. If these genes are known to have an activity in common—such as a mitochondrial or neurological or immunological function—then this common feature prioritizes cellular processes and molecular mechanisms that could be causally involved in disease. Framing such causal hypotheses has been aided considerably by the knowledgebase of gene function, including activity levels, molecular mechanism and cellular function, which have been growing substantially and rapidly over recent years as a result of novel and higher throughput technologies.

Secondly, a GWAS would enable detection of genetic signals that ME/CFS shares with other diseases or traits. Methods ((57)) that compare GWAS summary statistics for ME/CFS and other traits are available to calculate the genetic correlations between them. Genetic signals for ME/CFS could be shared with other diseases just as autoimmune diseases (for example, rheumatoid arthritis, type 1 diabetes and autoimmune thyroid disease) share such signals and underlying mechanisms of disease (58).

Thirdly, a GWAS could aid stratification of ME/CFS subtypes. Despite their well-defined clinical diagnoses, complex diseases such as type 2 diabetes are caused by diverse molecular and cellular mechanisms (59) and this should also be expected of ME/CFS. Its underlying biological subtypes could eventually be detectable using methods that test for genetic effect heterogeneity (60).

Lastly, discovery of genetic factors for ME/CFS risk might be expected to improve how this disorder is perceived by health professionals and by society at large.

## Future Perspective

Genetics studies are the best way to understand the aetiology of ME/CFS, because of the causal nature of genetic associations. A large GWAS focused on discovering the biomolecular mechanisms of ME/CFS is urgently needed because no study on the genetics of ME/CFS yet has seen results repeated under replication. For an appropriately powered GWAS, at least 10^4^ participants are required, and an equal or greater number of controls. A strict *P* < 5 × 10^−8^ or 1 × 10^−8^ statistical significance threshold must also be applied to reduce the numerous false positive associations seen from the meta-analyses presented here.

Although recruiting thousands of people with ME/CFS—particularly severely affected individuals who are housebound or bedbound—is a challenging task, it will be essential to perform a GWAS using their samples if we are to understand the mechanisms of the disease. With case criteria refined using genetic findings it may then be possible to begin stratifying the disease into distinct subtypes each with a different causal mechanism and potentially a specific treatment.

## Electronic Database Information

People with ME/CFS can now register to participate in this project at https://www.decodeme.org.uk/

*Conflict of Interest statement*. None declared.

## Funding

The Medical Research Council [MC_UU_00007/15 to C.P.P.]; Action for ME and the Chief Scientist Office, Scotland [AME/CSO/18/01]; the Medical Research Council and National Institute for Health Research for funding the DecodeME GWAS project [MC_PC_20005].
